# Duplication and functional divergence of a calcium sensor in the Brassicaceae

**DOI:** 10.1093/jxb/eraa031

**Published:** 2020-01-28

**Authors:** Shea M Monihan, Courtney A Magness, Choong-Hwan Ryu, Michelle M McMahon, Mark A Beilstein, Karen S Schumaker

**Affiliations:** 1 School of Plant Sciences, University of Arizona, Tucson, AZ, USA; 2 University of Western Australia, Australia

**Keywords:** Brassicaceae, CALCINEURIN B-LIKE10, calcium sensor, functional divergence, gene duplication, salt tolerance

## Abstract

The presence of varied numbers of *CALCINEURIN B-LIKE10* (*CBL10*) calcium sensor genes in species across the Brassicaceae and the demonstrated role of *CBL10* in salt tolerance in *Arabidopsis thaliana* and *Eutrema salsugineum* provided a unique opportunity to determine if *CBL10* function is modified in different species and linked to salt tolerance. Salinity effects on species growth and cross-species complementation were used to determine the extent of conservation and divergence of *CBL10* function in four species representing major lineages within the core Brassicaceae (*A. thaliana*, *E. salsugineum*, *Schrenkiella parvula*, and *Sisymbrium irio*) as well as the first diverging lineage (*Aethionema arabicum*). Evolutionary and functional analyses indicate that *CBL10* duplicated within expanded lineage II of the Brassicaceae and that, while portions of *CBL10* function are conserved across the family, there are species-specific variations in *CBL10* function. Paralogous *CBL10* genes within a species diverged in expression and function probably contributing to the maintenance of the duplicated gene pairs. Orthologous *CBL10* genes diverged in function in a species-specific manner, suggesting that functions arose post-speciation. Multiple *CBL10* genes and their functional divergence may have expanded calcium-mediated signaling responses and contributed to the ability of certain members of the Brassicaceae to maintain growth in salt-affected soils.

## Teaser

Duplication of the *CBL10* calcium sensor within expanded lineage II of the Brassicaceae led to both conservation of and species-specific variations in CBL10 function across the family.

## Introduction

Calcium has emerged as an essential component of many signaling pathways in plants, underlying growth and development by linking perception of physiological and environmental cues to cellular responses. Specificity in signaling is achieved, in part, through an array of proteins that perceive changes in cytosolic calcium levels (calcium sensors). The potential for different calcium sensors to contribute to functional specificity is found in their diverse temporal and spatial expression patterns, different affinities for calcium, and range of target proteins.

In *Arabidopsis thaliana* (Arabidopsis), *CALCINEURIN B-LIKE10* (*AtCBL10*) encodes a calcium sensor that enables plants to grow in salt-affected soils by preventing the toxic accumulation of sodium ions in the cytosol (Quan *et al.*, 2007). Upon perception of changes in cytosolic calcium levels, AtCBL10 interacts with the SALT-OVERLY-SENSITIVE2 (AtSOS2) protein kinase to activate the AtSOS1 plasma membrane sodium/proton exchanger which transports sodium out of the cell using the energy stored in the proton gradient (Quan *et al.*, 2007; Lin *et al.*, 2009). Mutation of *AtCBL10* results in hypersensitivity when plants are grown in the presence of even low levels of salt (Quan *et al.*, 2007). Two *CBL10* genes have been found in a salt-tolerant relative of Arabidopsis, *Eutrema salsugineum* (*EsCBL10a* and *EsCBL10b*; Monihan *et al.*, 2019). A comparative study of *CBL10* function in Arabidopsis and *E. salsugineum* provided a starting point for understanding how duplication of a calcium sensor increased calcium-mediated signaling capacity in *E. salsugineum* and contributed to its salt tolerance. Reduced expression of *EsCBL10a* and *EsCBL10b* singly and in combination revealed that both genes function in the response of *E. salsugineum* to salt and probably have different functions (Monihan *et al.*, 2019). Both *EsCBL10* genes complement the *Atcbl10* salt-sensitive phenotype, indicating that there is conservation of *CBL10* function in the two species (Monihan *et al.*, 2019). When the genes were expressed in a salt-sensitive strain of *Saccharomyces cerevisiae* (yeast) with *SOS2* and *SOS1* from Arabidopsis or *E. salsugineum*, cells expressing *EsCBL10b* had the greatest growth on media with salt, while cells expressing *EsCBL10a* had the weakest growth relative to cells expressing *AtCBL10* (Monihan *et al.*, 2019). This result suggests that EsCBL10b strongly activates the SOS pathway while EsCBL10a does so only weakly. The different expression patterns of the *EsCBL10* genes provided further evidence that these two genes diverged in function. *EsCBL10b*, like *AtCBL10*, is expressed primarily in leaves, with very low expression in roots, while *EsCBL10a* is expressed in both leaves and roots (Monihan *et al.*, 2019). AtSOS3, another calcium sensor in the CALCINEURIN B-LIKE family in Arabidopsis (also known as AtCBL4), is expressed in roots and also functions in plant responses to salt (Liu and Zhu, 1998; Halfter *et al.*, 2000). The AtSOS3 and AtCBL10 proteins have non-overlapping roles during growth in the presence of salt (Quan *et al.*, 2007). *EsCBL10a*, but not *AtCBL10* or *EsCBL10b*, was able to complement the salt-sensitive *Atsos3* mutant phenotype, suggesting that EsCBL10a has a function not present in AtCBL10 or EsCBL10b (Monihan *et al.*, 2019). Together these results demonstrated that the duplication of *CBL10* in *E. salsugineum* resulted in two calcium sensors with both shared and distinct activities expanding the response of *E. salsugineum* to salt.

Arabidopsis and *E. salsugineum* belong to the Brassicaceae, a diverse family of plants containing both economically and scientifically important species (Beilstein *et al.*, 2006; Yang *et al.*, 2013). The number of sequenced Brassicaceae genomes coupled with its well-established phylogeny led us to analyze the extent of conservation and divergence of *CBL10* gene function in both evolutionary and genomic contexts. Specifically we determined if: (i) other species in the Brassicaceae have multiple *CBL10* genes and if they are descendants of the same duplication event that resulted in two genes in *E. salsugineum*; (ii) the functions of the orthologous genes have been retained; and (iii) paralogous genes within selected species diverged in function. These analyses in combination with assays of plant growth in the presence of salt were used to understand how changes in *CBL10* function may have contributed to Brassicaceae adaptation to soil salinity. A phylogenetic analysis of *CBL10* revealed that the duplication resulting in two genes in *E. salsugineum* probably occurred in expanded lineage II (to which *E. salsugineum* belongs) of the family and resulted in several species retaining orthologs of *EsCBL10a* and *EsCBL10b*. *CBL10* expression and function were examined in four species representing major lineages within the core Brassicaceae (*A. thaliana*, *E. salsugineum*, *Schrenkiella parvula*, and *Sisymbrium irio*) as well as the first diverging lineage (*Aethionema arabicum*) (Beilstein *et al.*, 2006). These analyses indicate that, while portions of *CBL10* function are conserved across the Brassicaceae, there are also variations in *CBL10* function that are specific to each species.

## Materials and methods

### Phylogenetic tree

Nucleotide sequences were identified using Basic Local Alignment Search Tool (BLAST) with the coding sequence of *A, thaliana CBL10* (*AtCBL10*; At4G33000.2) and a threshold E-score of the order of 1×10^–15^. The identified genes were used in a reciprocal BLAST against the Arabidopsis genome. Only those sequences that identified *AtCBL10* as the closest homolog were used to generate the phylogeny. Sequences for *Arabidopsis lyrata* (Hu *et al.*, 2011), *Boechera stricta* (Windsor *et al.*, 2006), *Brassica nigra*, *Brassica oleraceae* (Liu *et al.*, 2014), *Brassica rapa* (Wang *et al.*, 2011), *Camelina sativa* (Kagale *et al.*, 2014), *Capsella grandiflora* (Slotte *et al.*, 2013), *Capsella rubella* (Slotte *et al.*, 2013), *Carica papaya* (Ming *et al.*, 2008), *E. salsugineum* Shandong (Yang *et al.*, 2013), and *Raphanus raphanistrum* (Moghe *et al.*, 2014) were retrieved from the Phytozome database (www.phytozome.net). Sequences for *S. parvula* (Dassanayake *et al.*, 2011) were retrieved from the NCBI genome database, while sequences for *A. arabicum*, *Neslia paniculata*, *Leavenworthia alabamica*, and *S. irio* were provided by Dr Stephen Wright (University of Toronto; Haudry *et al.*, 2013; Slotte *et al.*, 2013), and a sequence for *Tarenaya hassleriana* (Cheng *et al.*, 2013) was retrieved from the Comparative Genomics (CoGe) platform (Lyons and Freeling, 2008; Lyons *et al.*, 2008). Sequences for *CBL10* from the *E. salsugineum* accessions Cracker Creek, and Yukon were obtained by PCR using primers designed to amplify *CBL10* from the Shandong accession. To strengthen the inference of the root for the gene tree within the Brassicaceae, outgroup sequences for *Populus euphratica* (Ma *et al.*, 2013), *Prunus mume* (Zhang *et al.*, 2012), *Durio zibethinus* (Teh *et al.*, 2017), *Theobroma cacao* (Argout *et al.*, 2011), and *Vitis vinifera* (Jaillon *et al.*, 2007) were obtained from the GenBank nucleotide database using the *CBL10* sequence from *C. papaya* as a query for a BLAST search (all matched with E-scores of the order of 1×10^–140^).

Exon boundaries for the Arabidopsis, *E. salsugineum*, *S. parvula*, *S. irio*, and *A. arabicum CBL10* genes were determined by comparing the genomic sequences with cDNAs generated by PCR [*AtCBL10* (Quan *et al.*, 2007); *EsCBL10a* and *EsCBL10b* (Monihan *et al.*, 2019); *S. parvula*, *S. irio*, and *A. arabicum CBL10* genes, see cloning strategy in the complementation assay section below]. For all other *CBL10* genes, exon boundaries were estimated after multiple sequence alignments to the experimentally determined annotations. Extensive length heterogeneity, indels, and low-complexity sequences within each of the eight introns resulted in poorly aligned sequences. The *CBL10* coding sequences from the nine exon regions were concatenated, aligned in MUSCLE 3.8.31 (Edgar, 2004) using translated amino acids, and analyzed using IQ-TREE 1.6.7 (Nguyen *et al.*, 2015) with standard model selection. Two sequences from *L. alabamica* failed the composition test, indicating statistically significant differences in composition of these two sequences relative to the rest of the alignment. The model selected by the Bayesian information criterion and the corrected Akaike information criterion was TIM3+F+I+G4, which was used in the subsequent likelihood tree search with support for branches estimated by 100 bootstrap replicates.

### Synteny analysis

The genomic regions of Arabidopsis containing CBL10 and *E. salsugineum* containing *EsCBL10a* and *EsCBL10b* were compared with 15 genomes (*A. arabicum*, *A. lyrata*, *B. stricta*, *B. nigra*, *B. oleraceae*, *B. rapa*, *C. sativa*, *C. grandiflora*, *C. rubella*, *C. papaya*, *L. alabamica*, *R. raphanistrum*, *S. parvula*, *S. irio*, and *T. hassleriana*) to identify collinear regions using the SynFind feature on CoGe (Lyons and Freeling, 2008; Lyons *et al.*, 2008). The genes located on the two identified collinear regions of the Arabidopsis genome were used as a query for a BLAST search to detect putative homologs in the genomes of the other species (Altschul *et al.*, 1990). The species tree shown in the synteny analysis is based on Beilstein *et al.* (2010) and Yang *et al.* (2013).

### Identification of transposable elements

RepeatMasker 4.0.6 was used to detect putative transposable elements in flanking regions of *CBL10/CBL10a* and *CBL10b* in Arabidopsis, *E. salsugineum*, *S. irio*, and *S. parvula* (A.F.A. Smit, R. Hubley, and P. Green; RepeatMasker at http://repeatmasker.org).

### Plant growth

To determine the salt tolerance of each species, seeds of Arabidopsis, *E. salsugineum* Shangdong, *S. parvula*, *S. irio*, and *A. arabicum* were sown on SunGro Sunshine LC1 soil mix (SunGrow Horticulture) and stratified for 4 d at 4 °C in the dark to break dormancy. After cold treatment, plants were transferred to a growth chamber at 21 °C under a 16 h light/8 h dark photoperiod with light provided by Phillips F32T8/TL841 bulbs (135 µmol m^−2^ s^−1^) and watered every 3 d with 0.25× Hoagland’s solution (Hoagland and Arnon, 1938) with cobalt chloride in place of cobalt nitrate. When true leaves developed, plants were treated with increasing salt (sodium chloride, NaCl) in 50 mM increments every 3 d until the indicated, final concentration was reached. Three weeks after the start of treatment, photographs were taken and the fresh weight of aerial tissue was recorded. Comparisons of growth were performed by analyzing the ratio of species growth in the absence and presence of salt at each NaCl concentration using Friedman’s non-parametric, two-way ANOVA and Tukey’s honestly significant difference (HSD) tests. Statistical significance was assigned at *P*≤0.05.

### Complementation assays

*CBL10* gene function was analyzed by expressing each gene in the *Atcbl10* T-DNA insertion mutant (Arabidopsis Biological Resource Center, SALK_056042; Monihan *et al.*, 2016) and the *Atsos3* ethyl methanesulfonate mutant (Dr Jian-Kang Zhu; Purdue University; Liu and Zhu, 1997). Full-length coding sequences without a stop codon were amplified from cDNA and cloned into pGEM-T Easy (Promega). All PCR amplifications were performed using Phusion High-Fidelity DNA Polymerase (ThermoFisher Scientific); primer sequences are provided in Supplementary Table S1 at *JXB* online. Because of the high degree of similarity between the *S. parvula* transcripts, forward primers were designed to anneal to the 5'-untranslated region (UTR) to enrich for a specific *CBL10* gene; primer sequences are provided in Supplementary Table S1. The *S. parvula* PCRs were then used as a template in a second reaction with primers that anneal to the coding sequence for cloning into pGEM-T Easy (Promega). All genes were digested with *Xho*I and *Bam*HI, and subcloned into the corresponding site of the plant binary vector pEZT-NL (Drs Sean Cutler and David W. Ehrhardt; Carnegie Institution of Washington) downstream of the *Cauliflower mosaic virus* (CaMV) 35S promoter. *Agrobacterium tumefaciens* LBA4404 containing the binary vector was used to transform *Atcbl10* and *Atsos3* via the floral dip method (Clough and Bent, 1998). T_1_ seed was germinated on soil for 1.5 weeks and then sprayed three times with 100 mg l^–1^ Basta (Rely 200 Herbicide; Bayer Crop Science) at 3 d intervals. T_1_ lines with antibiotic resistance were subsequently transferred to pots and grown to collect T_2_ seed. Single insertion lines were identified by screening T_2_ seed on 0.5× MS medium [Murashige and Skoog medium; PhytoTechnology Laboratories containing 2.5 mM MES, 2% sucrose (w/v), and 1% agar (w/v) (A8678; Sigma), pH 5.7 (adjusted with potassium hydroxide)] and 7.5 mg l^–1^ glufosinate ammonium (Santa Cruz Biotechnology). Lines with 75% resistance were selected. Homozygous lines were identified by screening T_3_ seed on MS plates with glufosinate ammonium and selecting lines with 100% resistance. Gene-specific primers were used to confirm the identity and expression of all transgenes (Supplementary Fig. S1).

To monitor seedling growth, transgenic Arabidopsis seeds were sown on 0.5× MS medium. Plates with seed were incubated at 4 °C in the dark for 2 d to break dormancy and transferred to a growth chamber at 21 °C under a 16 h light/8 h dark photoperiod with light provided by Phillips F32T8/TL841 bulbs (135 µmol m^−2^ s^−1^). For salt assays, seeds were germinated on medium without NaCl for 4 d, after which seedlings were transferred to medium without or with the indicated concentration of NaCl. NaCl concentrations were chosen for maximal differences in growth between the wild type and mutants. After 10 d of treatment, photographs were taken and seedling fresh weight was measured to quantify growth.

### Transcript analysis

To determine expression patterns of the *CBL10* genes, leaves and roots of 11-day-old seedlings grown on 0.25× MS medium were collected. RNA was isolated using the NucleoSpin RNA extraction kit (Macherey-Nagel) and used to synthesize cDNA (M-MLV Reverse Transcriptase; Promega). All PCRs were performed using recombinant Taq polymerase (Invitrogen) and were analyzed and compared in the linear range of the amplification (e.g. 23 cycles for *Actin* and 28 cycles for *CBL10*); primer sequences are provided in Supplementary Table S1.

### Yeast salt screens

To monitor the ability of the CBL10 proteins to activate the SOS pathway, *Saccharomyces cerevisiae* strain AXT3K (*ena1*::*HIS3*::*ena4*, *nha1*::*LEU2*, and *nhx1*::*KanMX4*; Quintero *et al.*, 2002) containing the pYPGE15 plasmid with *AtSOS1* (Jarvis *et al.*, 2014) and the pFL32T plasmid with *AtSOS2* and *AtSOS3* was modified to express the *CBL10* genes. Plasmids containing *AtCBL10*, *EsCBL10a*, *EsCBL10b*, or without a *CBL10* gene were generated as previously described (Monihan *et al.*, 2019). The full-length coding sequences of *SpCBL10a*, *SpCBL10b-1*, *SpCBL10b-2*, *SiCBL10a*, *SiCBL10b*, and *AaCBL10* were amplified from cDNA (Phusion High-Fidelity DNA polymerase; ThermoFisher Scientific; primer sequences are provided in Supplementary Table S1), cloned into pGEM-T Easy (Promega), digested with *Xho*I and *Not*I, and subcloned into the corresponding site of the pDR195 vector (Dr Alonso Rodriguez-Navarro; Rentsch *et al.*, 1995). Because of the high degree of similarity between the *S. parvula* transcripts, forward primers were designed to anneal to the 5'-UTR to enrich for a specific *CBL10* gene (Supplementary Table S1). The *S. parvula* PCRs were then used as a template in a second reaction with primers that anneal to the coding sequence for cloning into pGEM-T Easy (Promega). The pDR195 plasmids were digested with *Age*I and *Not*I, and a fragment containing the *CBL10* gene was subcloned into the corresponding site of the pFL32T plasmid in place of *AtSOS3* to be expressed with *AtSOS2*. Transformed AXT3K cells were selected on synthetic dropout medium lacking both uracil and tryptophan (Clontech/TaKaRa) containing yeast nitrogen base without amino acids (VWR). Salt assays were carried out in alkali cation-free medium (AP; Rodriguez-Navarro and Ramos, 1984) containing 1 mM KCl with the designated concentrations of NaCl and cultured at 30 °C for 4 d.

### Yeast two-hybrid assays

To determine if EsCBL10a and the SpCBL10b proteins might complement *Atsos3* by interaction with a similar CBL-interacting protein kinase (CIPK), yeast-two hybrid assays were performed between both SpCBL10b proteins and the four EsCBL10a-interacting AtCIPK proteins. Cloning of *AtCBL10*, *EsCBL10a*, *EsCBL10b*, and *AtCIPK* genes was described previously (Monihan *et al.*, 2019). *SpCBL10b-1* and *SpCBL10b-2* genes were PCR amplified using Phusion High-Fidelity DNA Polymerase (ThermoFisher Scientific); primer sequences are provided in Supplementary Table S1. The PCR products were digested with *Eco*RI and *Bam*HI, and cloned into the corresponding site of the pGADT7 and pGBKT7 vectors (Clontech/TaKaRa) which allows for expression of the gene as a fusion protein with the GAL4 DNA activation domain (AD) or the GAL4 DNA-binding domain (BD), respectively. The pGADT7 clones were transformed into *S. cerevisiae* strain Y2HGold (Clontech/TaKaRa), while the pGBKT7 clones were transformed into *S. cerevisiae* strain Y187 (Clontech/TaKaRa). Yeast were mated and grown on synthetic defined medium (SD) minus leucine and tryptophan (SD-LW) to select for diploid yeast expressing both constructs. To determine interaction, serial dilutions of yeast colonies were grown on SD-LW and without histidine (H), and incubated for 5 d. Interaction is shown in only one orientation because the SpCBL10b proteins fused to the GAL4 BD self-activated, causing strains expressing only these fusion proteins to grow on all selection media, masking any interaction with the CIPK proteins.

### Statistical analysis

To determine significant differences in growth, experiments were organized and analyzed as a randomized complete block design with genotypes and salt concentrations as treatments, and individual experiments as replicates. Treatment effects were assessed using a full-factorial mixed-model ANOVA in JMP, Version 11 (SAS Institute; 1989–2007). In these analyses, treatments were considered fixed effects and replicates random effects. The normality of the distributions of all dependent variables was analyzed by examining a plot of the residuals from a full-factorial ANOVA of untransformed data. A Shapiro–Wilk test (Shapiro and Wilk, 1965) was performed to assess normality, and Bartlett’s (Bartlett, 1937) and Levene’s (Levene, 1960) tests were performed to evaluate the homogeneity of variance. Based on the pattern of distribution and the results of these tests, a non-parametric approach was used to analyze the data throughout. Data were rank transformed using Microsoft Excel (function: RANK) followed by an ANOVA and Tukey’s HSD test for multiple comparisons of means (Conover and Iman, 1981). The HSD values from rank-based ANOVA were then applied to the actual means for each measurement (i.e. not the ranks used in ANOVA). Statistical significance was assigned at *P*≤0.05 throughout, and all tests of significance were two sided.

## Results

### *CBL10* duplicated within expanded lineage II of the Brassicaceae

Previous studies have shown that there are two CALCINEURIN B-LIKE10 (CBL10) calcium sensors in *E. salsugineum*, a salt-tolerant relative of Arabidopsis. Reduced expression of the duplicated *E. salsugineum CBL10* genes demonstrated that both genes function in the response of the plant to salt (Monihan *et al.*, 2019). Cross-species complementation assays demonstrated that the two *E. salsugineum* CBL10 proteins have both shared and distinct activities (Monihan *et al.*, 2019), suggesting that duplication of CBL10 increased calcium-mediated signaling capacity in *E. salsugineum*.

**Fig. 1.**
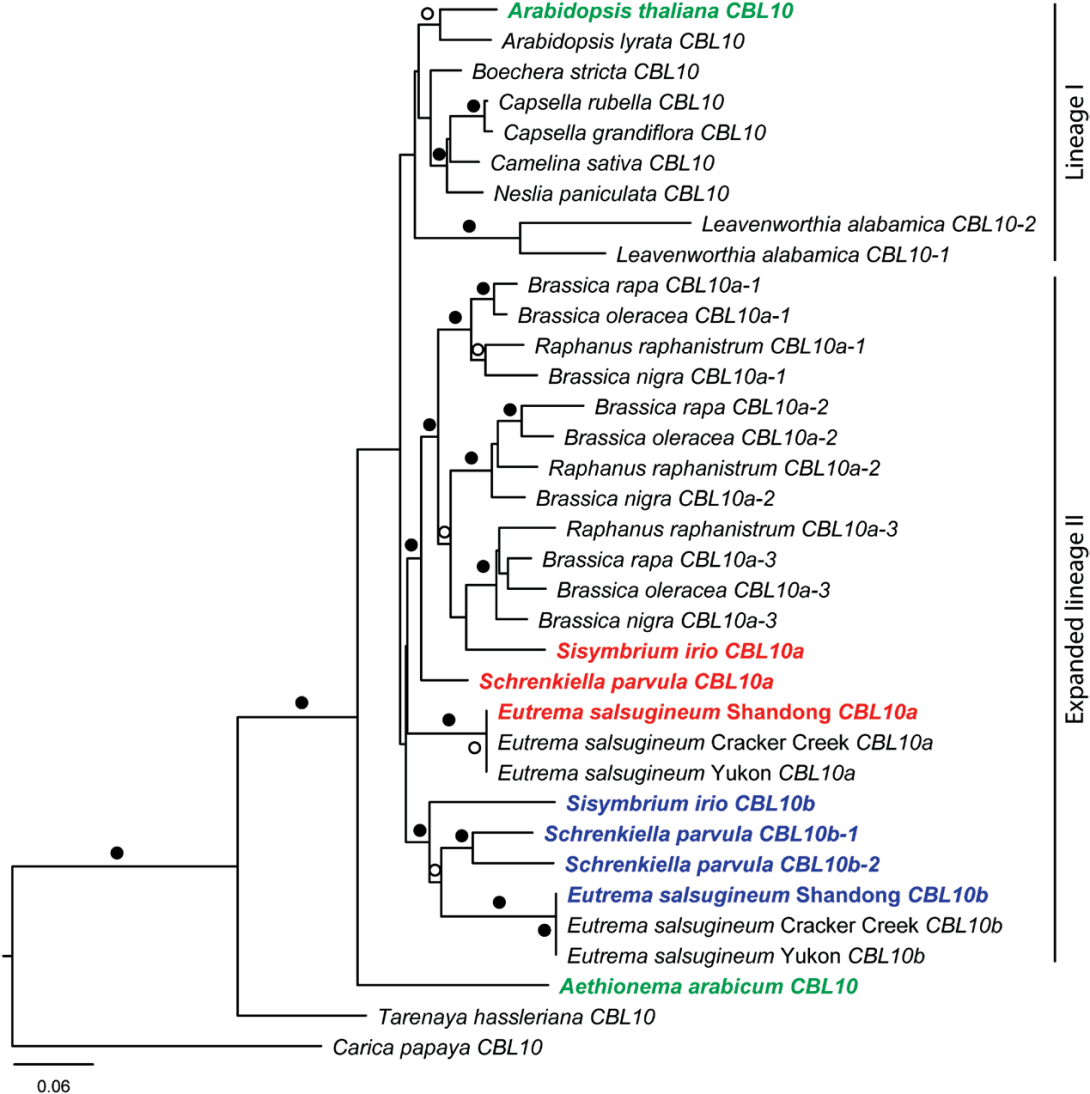
The duplication of *CBL10* probably occurred within expanded lineage II in the Brassicaceae. Exons from *CBL10* nucleotide sequences were aligned and analyzed using maximum likelihood. Sequences from *P. euphratica*, *P*. *mume*, *D. zibethinus*, *T. cacao*, and *V. vinifera* were included to provide additional data for the tree which was rooted on the *V. vinifera* sequence. Circles above branches represent the percentage of 100 bootstrap replicates that support the topology; closed circles, 90–100; open circles, 70–89. *CBL10* genes from five species were chosen for further analysis and color coded. Green, *CBL10* genes from two species with a single gene; red, *CBL10a* genes from three species with multiple genes; blue, *CBL10b* genes from three species with multiple genes. Species within lineage I and expanded lineage II are indicated.

**Fig. 2.**
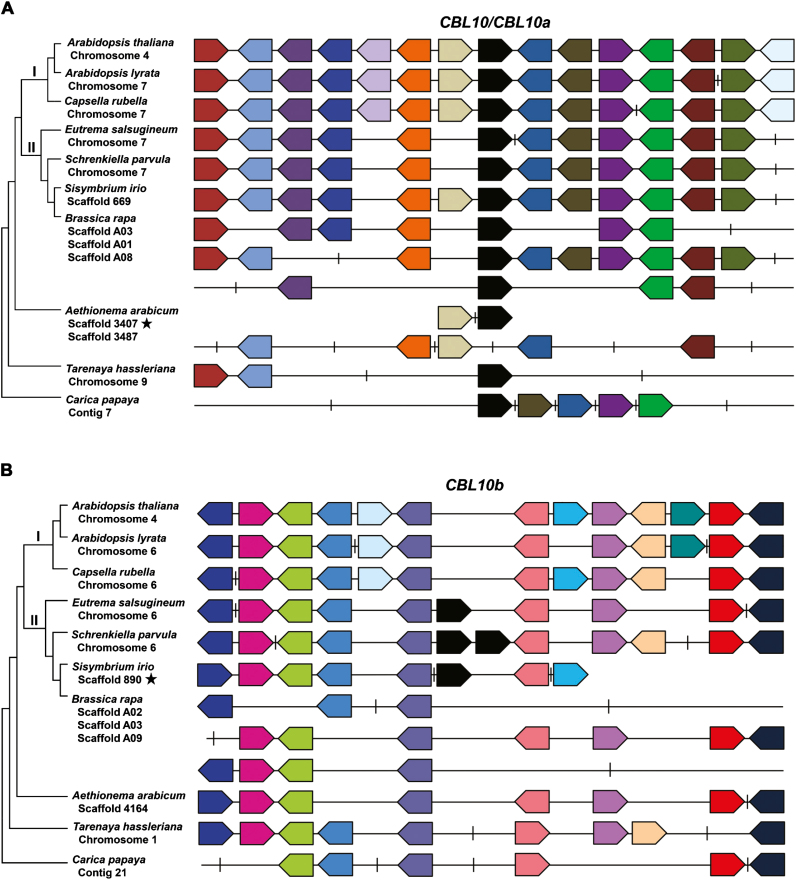
The genomic positions of *AtCBL10* and the *CBL10b* genes differ. The organismal phylogeny is shown with the species name and the chromosome, contig, or scaffold on which *CBL10* was identified. Stars, short scaffolds; horizontal line, genomic region (not drawn to scale); pentagons, genes. In Arabidopsis, *CBL10* is black and the genes on either side were identified and assigned a color. In the other species, *CBL10* genes were identified and the surrounding genes were colored based on ontology with genes from Arabidopsis. Vertical lines, presence of genes not syntenic to those in Arabidopsis. Tandem duplicates of the flanking genes were collapsed to one gene/pentagon to simplify the figure. (a) Regions syntenic to *AtCBL10* and *EsCBL10a*. (b) Regions syntenic to *EsCBL10b*.

To understand CBL10’s role in calcium-mediated signaling in an evolutionary context and determine when in the evolutionary history of the Brassicaceae the *CBL10* duplication arose, *CBL10* sequences were identified from species within and outside of the Brassicaceae and a phylogeny was inferred based on maximum likelihood. Of eight species examined within lineage I of the Brassicaceae, seven have a single *CBL10* gene: *A. thaliana*, *A. lyrata*, *B. stricta*, *C. rubella*, *C. grandiflora*, *C. sativa*, and *N. paniculata* (Fig. 1). *Leavenworthia alabamica* was the only lineage I species examined, with multiple *CBL10* genes probably due to a whole-genome triplication event; however, only two *CBL10* genes were identified (Fig. 1; Haudry *et al.*, 2013). *CBL10* sequences from *B. rapa*, *B. nigra*, *B. oleracea*, *R. raphanistrum*, *S. irio*, and *S. parvula* (lineage II species) were compared with the *CBL10* genes in *E. salsugineum* (which belongs to expanded lineage II; Beilstein *et al.*, 2006; Yang *et al.*, 2013). *Sisymbrium irio*, *S. parvula*, and *E. salsugineum* have both *CBL10a* and *CBL10b* paralogs (Fig. 1), with an additional *CBL10b* gene in *S. parvula* due to a tandem duplication (Fig. 2; Dassanayake *et al.*, 2011; Oh *et al.*, 2014). *Raphanus raphanistrum* and all three Brassica species have three *CBL10a* genes, consistent with their whole-genome triplication events (Fig. 1; Lagercrantz and Lydiate, 1996; Moghe *et al.*, 2014), but no *CBL10b* genes. *Aethionema arabicum*, which is a member of the first diverging lineage of the Brassicaceae, contains only a single *CBL10* gene (Fig. 1). Outside of the Brassicaceae, *T. hassleriana* and *C. papaya*, members of the Cleomaceae and Caricaceae, respectively, both have a single *CBL10* gene (Fig. 1). Sequences from *P. euphratica*, *P*. *mume*, *D. zibethinus*, *T. cacao*, and *V. vinifera* were included to provide additional data for the tree which was rooted on the *V. vinifera* sequence (data not shown). The tree is consistent with a single duplication in the ancestor of lineage II, leading to all known *CBL10b* genes which form a strongly supported clade (Fig. 1). The Brassica and *R. raphanistrum CBL10* genes descended from a triplication in the ancestor of just those species, not from the duplication that led to *CBL10b*, indicating that the ancestor of the Brassica species and *R. raphanistrum* lost *CBL10b*. *Sisymbrium irio CBL10a* was placed with weak support among the Brassica species, suggesting that additional duplications (or other phenomena such as lineage sorting) must have occurred. While it is not possible to unequivocally determine the timing of the duplication due to low branch support, the presence of both *CBL10a* and *CBL10b* paralogs in multiple members of expanded lineage II and the absence of the paralogs in members of lineage I, *A. arabicum*, *T. hassleriana*, and *C. papaya*, suggest that the duplication of *CBL10* occurred within expanded lineage II of the Brassicaceae.

The region surrounding the *CBL10* sequence was compared to provide additional information about the origin of the genes. *EsCBL10a* is syntenic with *CBL10* genes from Arabidopsis, *A. lyrata*, both Capsella species, *B. stricta*, *L. alabamica*, *C. sativa*, *T. hassleriana*, and *C. papaya*, and the *CBL10a* genes from all Brassica species, *S. parvula*, and *S. irio* (select species shown in Fig. 2A). Synteny in *N. paniculata* and *R. raphanistrum* was difficult to determine because the *CBL10* sequences were found on short scaffolds. The fact that all *CBL10* genes from the Brassica species and from *L. alabamica* share synteny with *EsCBL10a* (data not shown) further supports the conclusion that these *CBL10* genes arose through independent whole-genome triplication events (Lagercrantz and Lydiate, 1996; Haudry *et al.*, 2013). The *CBL10* gene from *A. arabicum* was found on a short scaffold with insufficient sequence to assess synteny (scaffold 3407, Fig. 2A). A second scaffold in *A. arabicum* sharing synteny with Arabidopsis was detected, but no *CBL10* gene was identified (scaffold 3487, Fig. 2A). The genomic region syntenic to *EsCBL10b* was identified in all species; however, a *CBL10b* gene was only detected in *E. salsugineum*, *S. parvula*, and *S. irio* (Fig. 2B). Numerous putative transposable elements were found surrounding the *CBL10b* genes, suggesting that the duplication of *CBL10* might have been mediated by transposons (Supplementary Fig. S2). However, it was not possible to identify a single transposon associated with all three *CBL10b* genes. Taken together, these results suggest that transposons may have mediated the duplication of *CBL10* and that the insertion of *CBL10b* into a different chromosomal position took place before the divergence of expanded lineage II species within the Brassicaceae.

### Species in expanded lineage II are salt tolerant

**Fig. 3.**
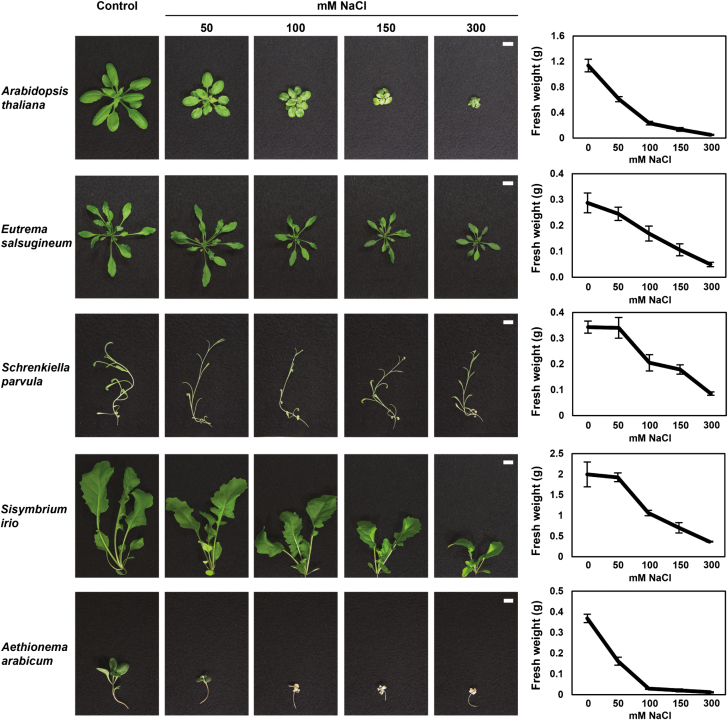
Species in expanded lineage II in the Brassicaceae are salt tolerant. Seeds from Arabidopsis, *E. salsugineum*, *S. parvula*, *S. irio*, and *A. arabicum* were germinated and grown on soil for 1 week and then treated with increasing NaCl in 50 mM increments every 3 d until the indicated final concentration was reached. Three weeks after the start of treatment, aerial portions of the plants were harvested, photographed, and weighed. Scale bar=1 cm for all images. The average fresh weight was graphed and ±SE is shown. One representative image of seven experiments is shown.

As a first step in linking *CBL10* function to salt tolerance within the Brassicaceae, five species were selected for further analyses. In addition to Arabidopsis and *E. salsugineum*, *S. parvula* and *S. irio* were chosen because of their close relationship to *E. salsugineum* and the presence of multiple *CBL10* genes in their genomes (Beilstein *et al.*, 2006; Fig. 3). Due to its position as a member of the first diverging group within the Brassicaceae and its more distant relationship with *E. salsugineum*, *A. arabicum* was also included in the analysis (Beilstein *et al.*, 2006; Fig. 3). The salt tolerance (FW of each species treated with increasing concentrations of salt) was measured. *Schrenkiella parvula*, *E. salsugineum*, and *S. irio* all maintained growth in concentrations of salt up to 300 mM, while growth of Arabidopsis and *A. arabicum* quickly decreased at concentrations as low as 100 mM (Fig. 3). Ratios of species growth in the absence and presence of salt at each salt concentration indicated that the salt tolerance of *S. parvula*, *E. salsugineum*, and *S. irio* was similar and distinct from the salt tolerance of Arabidopsis and *A. arabicum* which were similar (Supplementary Table S2). Because the increased salt tolerance of the expanded lineage II species analyzed might be due to an increase in the number of *CBL10* genes and/or a divergence in function of those genes, four assays (cross-species complementation of the *cbl10* and *sos3* mutants in Arabidopsis, expression analysis, and SOS pathway activation) were used to compare the activities of the genes.

### *CBL10* function is conserved in species across the Brassicaceae

**Fig. 4.**
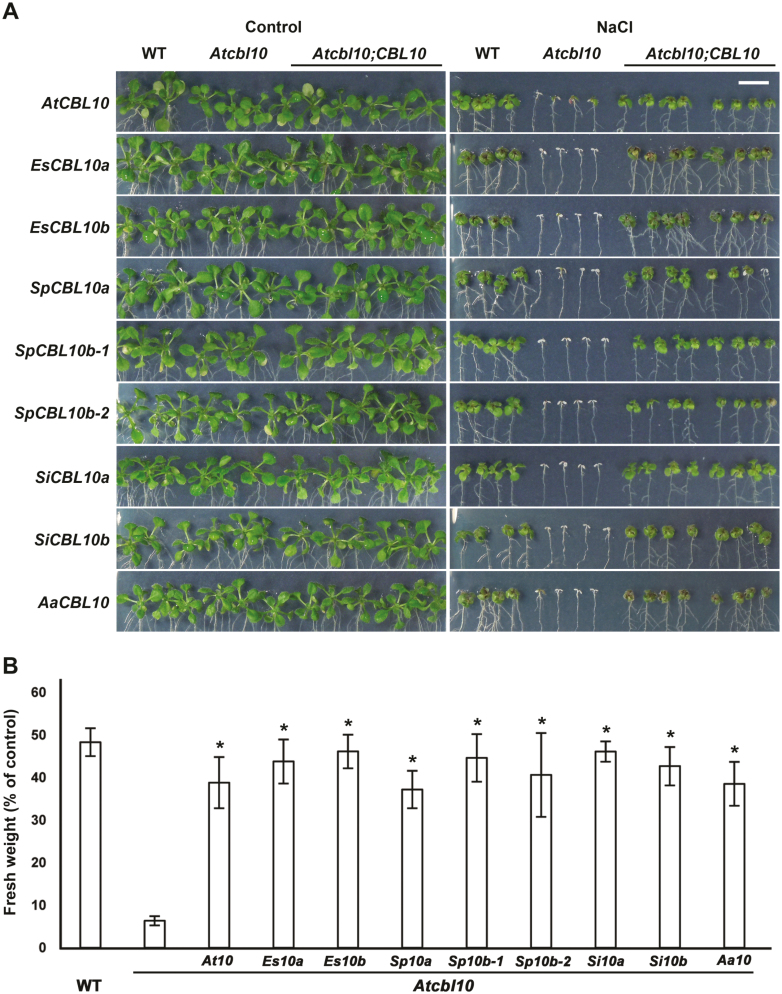
*CBL10* genes from all Brassicaceae species complement the *Atcbl10* salt-sensitive phenotype. *CBL10* genes from Arabidopsis (*AtCBL10*, *At10*), *E. salsugineum* (*EsCBL10a*, *Es10a*; and *EsCBL10b*, *Es10b*), *S. parvula* (*SpCBL10a*, *Sp10a*; *SpCBL10b-1*, *Sp10b-1*; and *SpCBL10b-2*, *Sp10b-2*), *S. irio* (*SiCBL10a*, *Si10a*; and *SiCBL10b*, *Si10b*), and *A. arabicum* (*AaCBL10*, *Aa10*) were expressed in the *Atcbl10* mutant, and growth in the absence (control) and presence of salt (125 mM NaCl) was monitored. For salt assays, seeds were germinated on medium without NaCl for 4 d, after which seedlings were transferred to medium without or with the indicated concentration of NaCl. After 10 d of treatment, photographs were taken and total seedling fresh weight was measured. (A) Photographs of the wild type (WT), *Atcbl10*, and *Atcbl10* expressing each *CBL10* gene (*Atcbl10;CBL10*). The scale bar (1 cm, upper right panel) shows the magnification for all images. (B) Total seedling fresh weight was measured to quantify growth and is presented as a percentage of control. Data are means ±SE of at least 24 seedlings per genotype grown in three independent experiments. *Complementation of the Atcbl10 salt-sensitive phenotype (Tukey–Kramer HSD, *P*≤0.05).

The *CBL10* phylogenetic tree suggests that all the genes identified are homologs of *AtCBL10* and may share activities. To determine if there is conservation of *CBL10* function throughout the Brassicaceae, the *CBL10* genes from *S. parvula*, *S. irio*, and *A. arabicum* were expressed in the Arabidopsis *cbl10* mutant (*Atcbl10*). Our studies have shown that introns are necessary for full expression and function of *AtCBL10* when using the native promoter, but can lead to alternative splicing. To avoid alternative splicing of the *CBL10* genes under study and to ensure strong expression of their coding sequences, the CaMV 35S constitutive promoter was used for these cross-species complementation assays. The salt tolerance of four independently transformed, single insertion, homozygous lines was assessed for each gene. All of the tested *CBL10* genes complemented the *Atcbl10* salt-sensitive phenotype, indicating that there is conservation of *CBL10* function throughout the Brassicaceae (Fig. 4).

### *CBL10* function diverged in a species-specific manner in the Brassicaceae

**Fig. 5.**
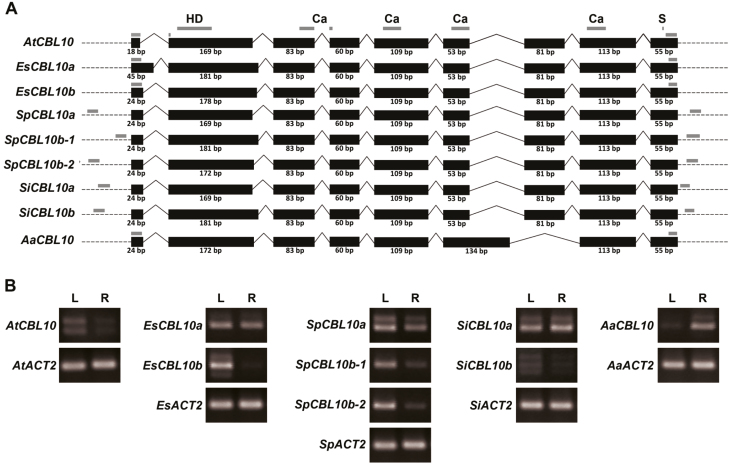
Expression of the *CBL10a* and *CBL10b* genes differs. (a) Transcript structure of the *CBL10* genes from Arabidopsis (*AtCBL10*), *E. salsugineum* (*EsCBL10a* and *EsCBL10b*), *S. parvula* (*SpCBL10a*, *SpCBL10b-1*, and *SpCBL10b-2*), *S. irio* (*SiCBL10a* and *SiCBL10b*), and *A. arabicum* (*AaCBL10*). Black boxes, exons (size indicated below in bp); solid lines, introns (not drawn to scale); dotted lines, untranslated regions; gray boxes, primer annealing sites. Protein domains important for the function of AtCBL10 are indicated above the *AtCBL10* transcript. HD, hydrophobic domain; Ca, calcium-binding domains; S, serine phosphorylation site. (b) *CBL10* transcript accumulation in leaves (L) and roots (R) of 11-day-old seedlings grown on 0.25× MS medium. *ACTIN2* (*ACT2*), loading control. One representative image of three replicates is shown.

It has previously been shown that the *CBL10* genes in *E. salsugineum* have different expression patterns. Like *AtCBL10*, *EsCBL10b* is expressed predominately in aerial tissue, whereas *EsCBL10a* is expressed throughout *E. salsugineum* (Monihan *et al.*, 2019). To determine if *CBL10* expression correlates with protein activity across the Brassicaceae, RNA was isolated from shoots and roots of each species and transcript accumulation was monitored. The exon–intron structure of the *CBL10* transcripts was very similar, and protein domains known to be important for AtCBL10 function were present in all transcripts (Fig. 5). Expression patterns correlated with the phylogenetic relationship of the genes in lineage I and expanded lineage II species; *CBL10b* transcripts were high in leaves (similar to *AtCBL10*) while *CBL10a* transcripts accumulated in both leaves and roots (Fig. 5). The expression pattern of the *CBL10* gene from *A. arabicum* was opposite to what was seen for the *CBL10b* and *AtCBL10* genes; the transcript was high in roots (Fig. 5).

**Fig. 6.**
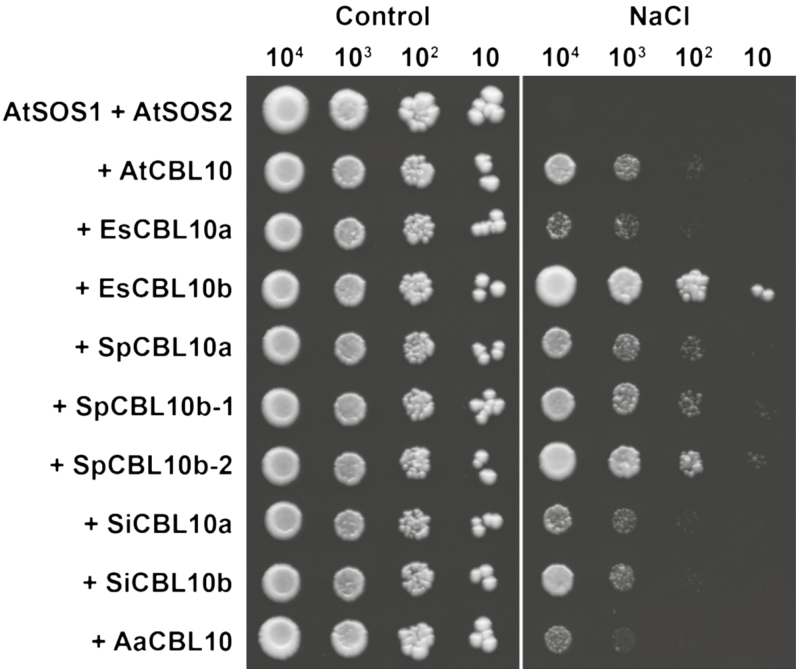
SOS pathway activation is greatest with EsCBL10b. A salt-sensitive strain of *S. cerevisiae* (AXT3K, *Δena1-4Δnha1Δnhx1*) was transformed with *SOS1* and *SOS2* from Arabidopsis in combination with *CBL10* from Arabidopsis (*AtCBL10*, *At10*), *E. salsugineum* (*EsCBL10a*, *Es10a*; and *EsCBL10b*, *Es10b*), *S. parvula* (*SpCBL10a*, *Sp10a*; *SpCBL10b-1, Sp10b-1*; and *SpCBL10b-2*, *Sp10b-2*), *S. irio* (*SiCBL10a*, *Si10a*; and *SiCBL10b*, *Si10b*), and *A. arabicum* (*AaCBL10*, *Aa10*). Serial decimal dilutions of yeast cells were spotted onto control medium or medium containing 125 mM NaCl. Two independently transformed colonies were assayed in three biological replicates; one representative image is shown.

AtCBL10 has been shown to activate the SOS pathway which functions to prevent the toxic accumulation of sodium in the cytoplasm (Quan *et al.*, 2007; Lin *et al.*, 2009). In *E. salsugineum*, differences in CBL10 activation of the pathway were observed; EsCBL10b strongly activated the Arabidopsis and *E. salsugineum* SOS pathways, while EsCBL10a showed only weak activation (Monihan *et al.*, 2019). To determine if the CBL10 proteins from *S. irio*, *S. parvula*, and *A. arabicum* function in the SOS pathway, each protein was expressed in a salt-sensitive strain of yeast (AXT3K) along with the Arabidopsis SOS2 protein kinase (AtSOS2) and SOS1 Na^+^/H^+^ exchanger (AtSOS1), and growth in the presence of salt was assessed as an indication of pathway activity. All CBL10 proteins activated the SOS pathway, but differences in the level of activation were observed. EsCBL10b had the greatest activity followed by the SpCBL10b and SpCBL10a proteins which all had greater activity than AtCBL10 (Fig. 6). The activities of SiCBL10a and SiCBL10b were similar to that of AtCBL10, while EsCBL10a and AaCBL10 had the weakest activity (Fig. 6).

**Fig. 7.**
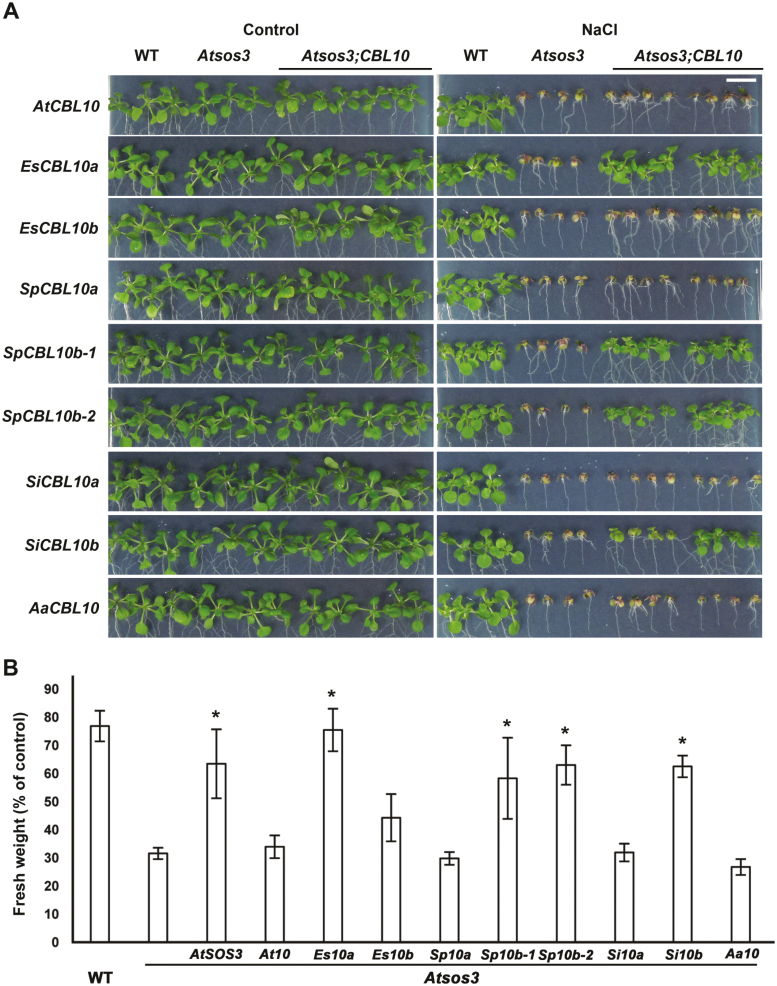
*EsCBL10a* and the *CBL10b* genes from *S. parvula* and *S. irio* complement the *Atsos3* salt-sensitive phenotype. *CBL10* genes from Arabidopsis (*AtCBL10*, *At10*), *E. salsugineum* (*EsCBL10a*, *Es10a*; and *EsCBL10b*, *Es10b*), *S. parvula* (*SpCBL10a*, *Sp10a*; *SpCBL10b-1*, *Sp10b-1*; and *SpCBL10b-2*, *Sp10b-2*), *S. irio* (*SiCBL10a*, *Si10a*; and *SiCBL10b*, *Si10b*), and *A. arabicum* (*AaCBL10*, *Aa10*) were expressed in the *Atsos3* mutant, and growth in the absence (control) and presence of salt (75 mM NaCl) was monitored. For salt assays, seeds were germinated on medium without NaCl for 4 d, after which seedlings were transferred to medium without or with the indicated concentration of NaCl. After 10 d of treatment, photographs were taken and total seedling fresh weight was measured. (A) Photographs of the wild type (WT), *Atsos3*, and *Atsos3* expressing each *CBL10* gene (*Atsos3;CBL10*). The scale bar (1 cm, upper right panel) shows the magnification for all images. (B) Total seedling fresh weight was measured to quantify growth and is presented as a percentage of the control. Data are means ±SE of at least 24 seedlings per genotype grown in three independent experiments. *Complementation of the *Atsos3* salt-sensitive phenotype (Tukey–Kramer HSD, *P*≤0.05).

EsCBL10a, but not AtCBL10 or EsCBL10b, can complement the salt-sensitive phenotype of the Arabidopsis *sos3* mutant (*Atsos3*), suggesting that EsCBL10a has a distinct function (Monihan *et al.*, 2019). To determine when this function arose within the Brassicaceae, the *S. irio*, *S. parvula*, and *A. arabicum CBL10* genes were expressed in *Atsos3* downstream of the CaMV 35S promoter and the salt tolerance of five independently transformed, single insertion, homozygous lines was determined. Four of the nine genes examined complemented *Atsos3*: *EsCBL10a*, *SpCBL10b-1*, *SpCBL10b-2*, and *SiCBL10b* (Fig. 7). These results indicate that at least one gene from each expanded lineage II species can perform a function that complements the *Atsos3* salt-sensitive phenotype.

**Fig. 8.**
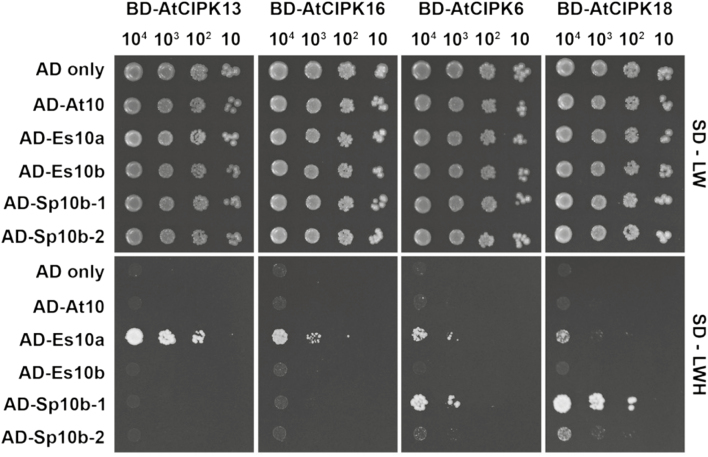
The SpCBL10b proteins interact with two of the four EsCBL10a-interacting CIPKs. CBL10 proteins from Arabidopsis (AtCBL10, A10), *E. salsugineum* (EsCBL10a, Es10a; and EsCBL10b, Es10b), and *S. parvula* (SpCBL10b-1, Sp10b-1; and SpCBL10b-2, Sp10b-2) were fused to the GAL4 activation domain (AD) and interaction with the Arabidopsis CIPKs fused to the GAL4 binding domain (BD) was assessed using yeast two-hybrid assays. Serial decimal dilutions of diploid yeast harboring both constructs were spotted onto synthetic defined media (SD) minus leucine (L) and tryptophan (W), or minus LW and histidine (H). Two independently mated colonies were assayed in two biological replications; one representative image is shown.

The ability of *EsCBL10a* to complement *Atsos3* and its weak ability to activate Arabidopsis and *E. salsugineum* SOS2 and SOS1 in yeast suggested that it might perform its functions with a different kinase (Monihan *et al.*, 2019). A yeast two-hybrid screen was performed with 26 kinases from the CIPK family to which AtSOS2 belongs. Four CIPKs (AtCIPK13, AtCIPK16, AtCIPK6, and AtCIPK18) were identified as interacting specifically with EsCBL10a but not AtCBL10 or EsCBL10b (Monihan *et al.*, 2019). To determine if the CBL10b proteins from *S. parvula* might complement *Atsos3* through a mechanism similar to EsCBL10a, interaction was tested between the SpCBL10b proteins and the four EsCBL10a-interacting CIPKs. SpCBL10b-1 interacted with two of the kinases, AtCIPK6 and AtCIPK18, while SpCBL10b-2 only interacted with AtCIPK18 (Fig. 8). In the opposite orientation, the *S. parvula* proteins self-activated, masking any interaction with the CIPK proteins, so interaction is only shown for the CIPK proteins fused to the GAL4 BD and the CBL10 proteins fused to the GAL4 AD.

## Discussion

### Cross-species analysis sheds light on the evolution of *CBL10* duplication in the Brassicaceae

**Fig. 9.**
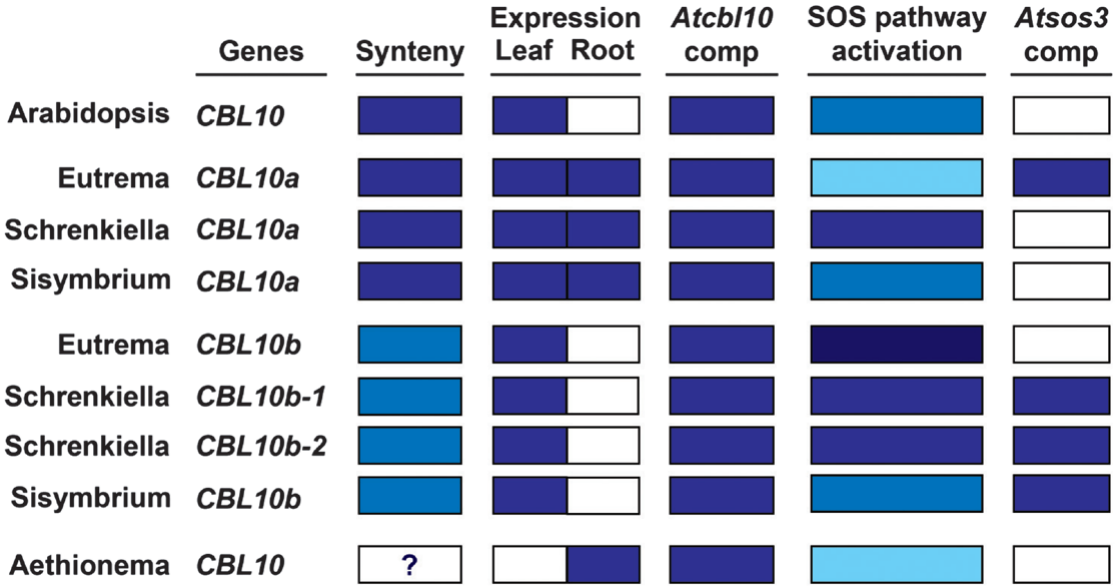
*CBL10* function diverged in a species-specific manner. Synteny, genes with the same color box are syntenic (white box indicates unknown synteny; Fig. 2). Expression, presence of *CBL10* transcript in leaves and roots (blue, present; white, absent; Fig. 5). *Atcbl10* comp, ability to complement the *Atcbl10* salt-sensitive phenotype (blue, complements; white, does not complement; Fig. 4). SOS pathway activation, ability to activate the Arabidopsis SOS pathway in yeast (strongest, strong, medium, and weak refer to the level of yeast growth correlating with strength of activation of the SOS pathway; Fig. 6). *Atsos3* comp, ability to complement the *Atsos3* salt-sensitive phenotype (blue, complements; white, does not complement; Fig. 7).

All of the *CBL10* genes examined complemented the *Atcbl10* salt-sensitive phenotype, indicating that at least a portion of *CBL10* function is conserved throughout the Brassicaceae (Figs 4, 9). This is consistent with studies of *CBL10* genes in *Poplar trichocarpa* and *Solanum lycopersicum* which were also able to complement *Atcbl10*, indicating that some of *CBL10* function is conserved outside of the Brassicaceae (Tang *et al.*, 2014; Egea *et al.*, 2018). However, within the Brassicaceae, species-specific differences in *CBL10* expression and function were detected.

The absence of the *CBL10a* and *CBL10b* paralogs in members of lineage I, *A. arabicum*, *T. hassleriana*, and *C. papaya* in combination with the presence of *CBL10a* and *CBL10b* paralogs in multiple members of lineage II suggest that the duplication of *CBL10* occurred within expanded lineage II of the Brassicaceae (Fig. 1). Lack of variation in the C-termini of the *CBL10* nucleotide sequences (containing the four highly conserved EF-hand calcium-binding domains) reduced phylogenetic resolution, precluding assignment of the duplication to a specific branch of the tree.

At least one gene from each species within expanded lineage II has the ability to complement the *Atsos3* salt-sensitive phenotype (Figs 7, 9). Whether the ability to complement *Atsos3* evolved prior to or after subsequent speciation in the expanded lineage II species sampled remains less clear; however, there is support for the changes occurring after speciation. The ability of EsCBL10a to complement *Atsos3* resides in the hydrophobic domain (Monihan *et al.*, 2019). Conservation of amino acids in this domain in proteins that do not complement (AtCBL10, EsCBL10b, SpCBL10a, SiCBL10a, and AaCBL10) and variation in proteins that do (EsCBL10a, SpCBL10b-1, SpCBL10b-2, and SiCBL10b) suggests that sequences changed post-*CBL10* duplication and after speciation (Supplementary Fig. S3). Additional studies will be required to determine if the other CBL10b proteins that complement *Atsos3* do so because of changes in this or another region of the protein.

With an increase in sequenced genomes and tools to analyze those genomes, questions have emerged regarding how genomic context influences which gene changes function after a duplication event. To determine if the change in function is equally likely in both genes or more likely in the gene inserted into a new genomic position, the ratio of non-synonymous (dN) to synonymous (dS) substitutions (dN/dS) is often calculated. Studies have generally concluded that the duplicated gene in the new genomic position often has the faster rate of change and, as a result, is the one most likely to change function (Han *et al.*, 2009; Dewey, 2011; Pegueroles *et al.*, 2013; Rosello and Kondrashov, 2014). In keeping with these findings, the *CBL10* genes in *S. parvula* and *S. irio* inserted into a new genomic position and acquired a function that complements *Atsos3*. However, *E. salsugineum* appears to be an exception to this generalization. EsCBL10a, which remained in the original genomic position, acquired the ability to complement *Atsos3*, while EsCBL10b, which inserted into a new genomic position, never acquired the function or acquired the function and subsequently lost it. In addition to influencing protein function, duplication and insertion of a gene into a new genomic context can lead to regulation by different *cis*-acting elements, suggesting that the gene that changes genomic position is more likely to have a different expression pattern (Flagel and Wendel, 2009; Pegueroles *et al.*, 2013). Our current data do not support this model because the *CBL10b* genes inserted into a new genomic position yet they share a similar expression pattern with *AtCBL10*, while the *CBL10a* genes, which remained in the original genomic position, appear to have expanded expression into roots. Analysis of expression patterns from additional species will be needed to establish the major expression pattern.

### Cross-species analysis sheds light on the molecular mechanism underlying *CBL10* duplication in the Brassicaceae

Transposable elements have been found to replicate and integrate into genomes, leading to duplication of genes (Xiao *et al.*, 2008; Flagel and Wendel, 2009; Panchy *et al.*, 2016). Because numerous transposable elements were found surrounding the *CBL10b* genes in the species studied (Supplementary Fig. S2), replicative transposition by transposable elements is a likely mechanism underlying the duplication of *CBL10*. *CBL10* duplication as a result of a polyploidization event is unlikely because no whole-genome duplication event is known to have occurred at the base of expanded lineage II and because, in the *CBL10*-containing regions of the genomes of species studied, *CBL10* is the only duplicated gene (Fig. 2). Because the *CBL10b* genes identified contain introns and are located on a different chromosome from the *CBL10a* genes, the duplication probably did not arise through a retroduplication or an unequal crossover event.

In Arabidopsis, the CBL calcium sensors (10 members) and the CIPK protein kinases (26 members) form signaling networks that link changes in cytosolic calcium levels to physiological responses (Luan, 2009). The weak ability of EsCBL10a to activate the SOS pathway suggested that it might function with a CIPK protein other than SOS2 (CIPK24) to complement *Atsos3* (Fig. 6; Monihan *et al.*, 2019). Using yeast two-hybrid assays, four CIPK proteins that interact with EsCBL10a but not EsCBL10b or AtCBL10 were identified (Monihan *et al.*, 2019). To begin to understand if EsCBL10a and the SpCBL10b proteins complement *Atsos3* through a similar mechanism, yeast two-hybrid assays were used to examine interaction between the SpCBL10b proteins and the four EsCBL10a-interacting CIPK proteins. Only AtCIPK18 interacted with EsCBL10a and both SpCBL10b proteins; however, a role for *AtCIPK18* in salt tolerance has not been reported and the *Atcipk18* mutant does not have a salt-sensitive phenotype (Fig. 8; data not shown). SpCBL10b-1 but not SpCBL10b-2 interacted with AtCIPK6 which has been shown to interact with AtSOS3 to recruit the potassium transporter, AtAKT2, to the plasma membrane (Fig. 8; Held *et al.*, 2011). The duplication appears to have expanded the range of CIPK interactions. Whether there is convergence on a specific CIPK remains an open question.

### There are multiple ways in which CBL10 might contribute to salt tolerance in the Brassicaceae

All three *Schrenkiella* genes complement *Atcbl10* (Figs 4, 9), but further examination of function revealed differences. In *E. salsugineum*, the *CBL10* genes have different functions; EsCBL10b strongly activates the SOS pathway while *EsCBL10a* has an unknown function that allows it to complement *Atsos3* (Monihan *et al.*, 2019). In *S. parvula*, a different pattern is observed; SpCBL10a, like EsCBL10b, strongly activates the SOS pathway although not as well (Figs 6, 9), and does not have a function that complements *Atsos3* (Figs 7, 9). *SpCBL10b-1* and *SpCBL10b-2* can both strongly activate the SOS pathway (like EsCBL10b although also not as well, Figs 6, 9) and complement the *Atsos3* salt-sensitive phenotype (like EsCBL10a, Figs 7, 9). These results suggest that having three *CBL10* genes with strong and overlapping functions has contributed to the ability of *S. parvula* to maintain growth in the presence of salt.

*Eutrema salsugineum* and *S. irio* have similar levels of growth in the presence of salt, the same number of *CBL10* genes, similar *CBL10* expression patterns, all *CBL10* genes in the two species complement *Atcbl10*, and one of the two *CBL10* genes in each species can complement *Atsos3* (Figs 1–7). However, activation of the SOS pathway revealed differences in how CBL10 functions within these species. In *E. salsugineum*, EsCBL10b strongly activates the pathway while EsCBL10a does so only weakly. In *S. irio*, SiCBL10a and SiCBL10b both moderately activate the SOS pathway, suggesting that there are multiple ways to acquire salt tolerance (Figs 6, 9). As was found in *E. salsugineum*, the *CBL10* genes in *S. irio* have diverged in function, probably increasing calcium-mediated signaling capacity and contributing to the ability of *S. irio* to grow in the presence of salt.

*Aethionema arabicum* is a member of the first diverging lineage of Brassicaceae and has only one *CBL10* gene (Fig. 1). *AaCBL10* may reside in a different genomic position; a scaffold syntenic to *AtCBL10* but lacking *AaCBL10* was identified in *A. arabicum* and *AaCBL10* was found on a short scaffold (Figs 2, 9). A different genomic location for *AaCBL10* might explain the altered expression of the transcript; while the *CBL10* genes in the other species studied are expressed in shoots and some also in roots, *AaCBL10* is the only gene expressed exclusively in roots (Figs 5, 9). Several results suggest that *AaCBL10* may not play a significant role in the ability of *A. arabicum* to grow in the presence of salt: (i) *A. arabicum* is sensitive to salt, suggesting that it has few active mechanisms to deal with the presence of salt in the soil (Fig. 3); (ii) while *AaCBL10* is functional in *Atcbl10*, it is probably complementing *AtCBL10*’s function in shoots (Figs 4, 9) and may not perform a similar function in *A. arabicum* roots; (iii) *AaCBL10* is unable to complement the salt-sensitive phenotype of *Atsos3* whose gene product functions in roots (Figs 7, 9; Quan *et al.*, 2007); and (iv) AaCBL10 only weakly activates the SOS pathway (Figs 6, 9). Because calcium is an important signaling molecule in many different plant processes, AaCBL10 may function in other calcium-mediated responses in *A. arabicum*—a mutational analysis will be required to uncover the role of *CBL10* in this species.

Taken together, results from this study have demonstrated that: the paralogous *CBL10* genes within a species diverged in expression and function probably contributing to the maintenance of the duplicated gene pairs in their genomes; orthologous *CBL10* genes have diverged in function in a species-specific manner, suggesting that the function of the genes was not established immediately after the duplication but after speciation; and that species studied with multiple *CBL10* genes are better able to grow in the presence of salt.

## Supplementary data

Supplementary data are available at *JXB* online.

Fig. S1. Specificity of *CBL10* gene expression in transgenic Arabidopsis.

Fig. S2. Transposable elements may have mediated the *CBL10* duplication.

Fig. S3. Differences in CBL10 function reside in the N-terminus.

Table S1. Primers.

Table S2. Ratio of species growth in the absence and presence of salt.

eraa031_suppl_Supplementary_Figures_S1-S3_and_Tables_S1-S2

## References

[CIT0001] Altschul SF, Gish W, Miller W, Myers EW, Lipman DJ 1990 Basic local alignment search tool. Journal of Molecular Biology 215, 403–410. 2231712 10.1016/S0022-2836(05)80360-2

[CIT0002] Argout X, Salse J, Aury JM, et al. 2011 The genome of *Theobroma cacao*. Nature Genetics 43, 101–108. 21186351 10.1038/ng.736

[CIT0003] Bartlett MS 1937 Properties of sufficiency and statistical tests. Proceedings of the Royal Society A: Mathematical and Physical Sciences 160, 268–282.

[CIT0004] Beilstein MA, Al-Shehbaz IA, Kellogg EA 2006 Brassicaceae phylogeny and trichome evolution. American Journal of Botany 93, 607–619. 21646222 10.3732/ajb.93.4.607

[CIT0005] Beilstein MA, Nagalingum NS, Clements MD, Manchester SR, Mathews S 2010 Dated molecular phylogenies indicate a Miocene origin for *Arabidopsis thaliana*. Proceedings of the National Academy of Sciences, USA 107, 18724–18728. 10.1073/pnas.0909766107 PMC2973009 20921408

[CIT0006] Cheng S, van den Bergh E, Zeng P, et al. 2013 The *Tarenaya hassleriana* genome provides insight into reproductive trait and genome evolution of crucifers. The Plant Cell 25, 2813–2830. 23983221 10.1105/tpc.113.113480 PMC3784582

[CIT0007] Clough SJ, Bent AF 1998 Floral dip: a simplified method for *Agrobacterium*-mediated transformation of *Arabidopsis thaliana*. The Plant Journal 16, 735–743. 10069079 10.1046/j.1365-313x.1998.00343.x

[CIT0008] Conover WJ, Iman RL 1981 Rank transformations as a bridge between parametric and nonparametric statistics. American Statistician 35, 124–129.

[CIT0009] Dassanayake M, Oh DH, Haas JS, et al. 2011 The genome of the extremophile crucifer *Thellungiella parvula*. Nature Genetics 43, 913–918. 21822265 10.1038/ng.889 PMC3586812

[CIT0010] Dewey CN 2011 Positional orthology: putting genomic evolutionary relationships into context. Briefings in Bioinformatics 12, 401–412. 21705766 10.1093/bib/bbr040 PMC3178058

[CIT0011] Edgar RC 2004 MUSCLE: multiple sequence alignment with high accuracy and high throughput. Nucleic Acids Research 32, 1792–1797. 15034147 10.1093/nar/gkh340 PMC390337

[CIT0012] Egea I, Pineda B, Ortíz-Atienza A, et al. 2018 The SlCBL10 calcineurin B-like protein ensures plant growth under salt stress by regulating Na^+^ and Ca^2+^ homeostasis. Plant Physiology 176, 1676–1693. 29229696 10.1104/pp.17.01605 PMC5813568

[CIT0013] Flagel LE, Wendel JF 2009 Gene duplication and evolutionary novelty in plants. New Phytologist 183, 557–564. 19555435 10.1111/j.1469-8137.2009.02923.x

[CIT0014] Halfter U, Ishitani M, Zhu JK 2000 The *Arabidopsis* SOS2 protein kinase physically interacts with and is activated by the calcium-binding protein SOS3. Proceedings of the National Academy of Sciences, USA 97, 3735–3740. 10.1073/pnas.040577697 PMC16309 10725350

[CIT0015] Han MV, Demuth JP, McGrath CL, Casola C, Hahn MW 2009 Adaptive evolution of young gene duplicates in mammals. Genome Research 19, 859–867. 19411603 10.1101/gr.085951.108 PMC2675974

[CIT0016] Haudry A, Platts AE, Vello E, et al. 2013 An atlas of over 90,000 conserved noncoding sequences provides insight into crucifer regulatory regions. Nature Genetics 45, 891–898. 23817568 10.1038/ng.2684

[CIT0017] Held K, Pascaud F, Eckert C, et al. 2011 Calcium-dependent modulation and plasma membrane targeting of the AKT2 potassium channel by the CBL4/CIPK6 calcium sensor/protein kinase complex. Cell Research 21, 1116–1130. 21445098 10.1038/cr.2011.50 PMC3193494

[CIT0018] Hoagland DR, Arnon DI 1938 The water-culture method for growing plants without soil. California Agricultural Experiment Station Circular 347, 1–39.

[CIT0019] Hu TT, Pattyn P, Bakker EG, et al. 2011 The *Arabidopsis lyrata* genome sequence and the basis of rapid genome size change. Nature Genetics 43, 476–481. 21478890 10.1038/ng.807 PMC3083492

[CIT0020] Jaillon O, Aury JM, Noel B, et al. 2007 The grapevine genome sequence suggests ancestral hexaploidization in major angiosperm phyla. Nature 449, 463–467. 17721507 10.1038/nature06148

[CIT0021] Jarvis DE, Ryu CH, Beilstein MA, Schumaker KS 2014 Distinct roles for SOS1 in the convergent evolution of salt tolerance in *Eutrema salsugineum* and *Schrenkiella parvula*. Molecular Biology and Evolution 31, 2094–2107. 24803640 10.1093/molbev/msu152

[CIT0022] Kagale S, Koh C, Nixon J, et al. 2014 The emerging biofuel crop *Camelina sativa* retains a highly undifferentiated hexaploid genome structure. Nature Communications 5, 3706. 10.1038/ncomms4706 PMC4015329 24759634

[CIT0023] Lagercrantz U, Lydiate DJ 1996 Comparative genome mapping in Brassica. Genetics 144, 1903–1910. 8978073 10.1093/genetics/144.4.1903 PMC1207737

[CIT0024] Levene H 1960 Robust tests for equality of variances. In: Olkin I, ed. Contributions to probability and statistics. Stanford, CA: Stanford University Press, 278–292.

[CIT0025] Lin H, Yang Y, Quan R, Mendoza I, Wu Y, Du W, Zhao S, Schumaker KS, Pardo JM, Guo Y 2009 Phosphorylation of SOS3-LIKE CALCIUM BINDING PROTEIN8 by SOS2 protein kinase stabilizes their protein complex and regulates salt tolerance in *Arabidopsis*. The Plant Cell 21, 1607–1619. 19448033 10.1105/tpc.109.066217 PMC2700523

[CIT0026] Liu J, Zhu JK 1997 An Arabidopsis mutant that requires increased calcium for potassium nutrition and salt tolerance. Proceedings of the National Academy of Sciences, USA 94, 14960–14964. 10.1073/pnas.94.26.14960 PMC25145 9405721

[CIT0027] Liu J, Zhu JK 1998 A calcium sensor homolog required for plant salt tolerance. Science 280, 1943–1945. 9632394 10.1126/science.280.5371.1943

[CIT0028] Liu S, Liu Y, Yang X, et al. 2014 The *Brassica oleracea* genome reveals the asymmetrical evolution of polyploid genomes. Nature Communications 5, 3930. 10.1038/ncomms4930 PMC4279128 24852848

[CIT0029] Luan S 2009 The CBL–CIPK network in plant calcium signaling. Trends in Plant Science 14, 37–42. 19054707 10.1016/j.tplants.2008.10.005

[CIT0030] Lyons E, Freeling M 2008 How to usefully compare homologous plant genes and chromosomes as DNA sequences. The Plant Journal 53, 661–673. 18269575 10.1111/j.1365-313X.2007.03326.x

[CIT0031] Lyons E, Pedersen B, Kane J, et al. 2008 Finding and comparing syntenic regions among Arabidopsis and the outgroups papaya, poplar, and grape: CoGe with rosids. Plant Physiology 148, 1772–1781. 18952863 10.1104/pp.108.124867 PMC2593677

[CIT0032] Ma T, Wang J, Zhou G, et al. 2013 Genomic insights into salt adaptation in a desert poplar. Nature Communications 4, 2797. 10.1038/ncomms3797 24256998

[CIT0033] Ming R, Hou S, Feng Y, et al. 2008 The draft genome of the transgenic tropical fruit tree papaya (*Carica papaya* Linnaeus). Nature 452, 991–996. 18432245 10.1038/nature06856 PMC2836516

[CIT0034] Moghe GD, Hufnagel DE, Tang H, Xiao Y, Dworkin I, Town CD, Conner JK, Shiu SH 2014 Consequences of whole-genome triplication as revealed by comparative genomic analyses of the wild radish *Raphanus raphanistrum* and three other Brassicaceae species. The Plant Cell 26, 1925–1937. 24876251 10.1105/tpc.114.124297 PMC4079359

[CIT0035] Monihan SM, Magness CA, Yadegari R, Smith SE, Schumaker KS 2016 Arabidopsis CALCINEURIN B-LIKE10 functions independently of the SOS pathway during reproductive development in saline conditions. Plant Physiology 171, 369–379. 26979332 10.1104/pp.16.00334 PMC4854721

[CIT0036] Monihan SM, Ryu CH, Magness CA, Schumaker KS 2019 Linking duplication of a calcium sensor to salt tolerance in *Eutrema salsugineum*. Plant Physiology 179, 1176–1192. 30606887 10.1104/pp.18.01400 PMC6393783

[CIT0037] Nguyen LT, Schmidt HA, von Haeseler A, Minh BQ 2015 IQ-TREE: a fast and effective stochastic algorithm for estimating maximum-likelihood phylogenies. Molecular Biology and Evolution 32, 268–274. 25371430 10.1093/molbev/msu300 PMC4271533

[CIT0038] Oh DH, Hong H, Lee SY, Yun DJ, Bohnert HJ, Dassanayake M 2014 Genome structures and transcriptomes signify niche adaptation for the multiple-ion-tolerant extremophyte *Schrenkiella parvula*. Plant Physiology 164, 2123–2138. 24563282 10.1104/pp.113.233551 PMC3982767

[CIT0039] Panchy N, Lehti-Shiu M, Shiu SH 2016 Evolution of gene duplication in plants. Plant Physiology 171, 2294–2316. 27288366 10.1104/pp.16.00523 PMC4972278

[CIT0040] Pegueroles C, Laurie S, Albà MM 2013 Accelerated evolution after gene duplication: a time-dependent process affecting just one copy. Molecular Biology and Evolution 30, 1830–1842. 23625888 10.1093/molbev/mst083

[CIT0041] Quan R, Lin H, Mendoza I, Zhang Y, Cao W, Yang Y, Shang M, Chen S, Pardo JM, Guo Y 2007 SCABP8/CBL10, a putative calcium sensor, interacts with the protein kinase SOS2 to protect Arabidopsis shoots from salt stress. The Plant Cell 19, 1415–1431. 17449811 10.1105/tpc.106.042291 PMC1913747

[CIT0042] Quintero FJ, Ohta M, Shi H, Zhu JK, Pardo JM 2002 Reconstitution in yeast of the Arabidopsis SOS signaling pathway for Na^+^ homeostasis. Proceedings of the National Academy of Sciences, USA 99, 9061–9066. 10.1073/pnas.132092099 PMC124423 12070350

[CIT0043] Rentsch D, Laloi M, Rouhara I, Schmelzer E, Delrot S, Frommer WB 1995 NTR1 encodes a high affinity oligopeptide transporter in Arabidopsis. FEBS Letters 370, 264–268. 7656990 10.1016/0014-5793(95)00853-2

[CIT0044] Rodríguez-Navarro A, Ramos J 1984 Dual system for potassium transport in *Saccharomyces cerevisiae*. Journal of Bacteriology 159, 940–945. 6384187 10.1128/jb.159.3.940-945.1984 PMC215750

[CIT0045] Roselló OPI, Kondrashov FA 2014 Long-term asymmetrical acceleration of protein evolution after gene duplication. Genome Biology and Evolution 6, 1949–1955 25070510 10.1093/gbe/evu159 PMC4159008

[CIT0046] Shapiro SS, Wilk MB 1965 An analysis of variance test for normality (Complete Samples). Biometrika 52, 591.

[CIT0047] Slotte T, Hazzouri KM, Ågren JA, et al. 2013 The *Capsella rubella* genome and the genomic consequences of rapid mating system evolution. Nature Genetics 45, 831–835. 23749190 10.1038/ng.2669

[CIT0048] Tang RJ, Yang Y, Yang L, Liu H, Wang CT, Yu MM, Gao XS, Zhang HX 2014 Poplar calcineurin B-like proteins PtCBL10A and PtCBL10B regulate shoot salt tolerance through interaction with PtSOS2 in the vacuolar membrane. Plant, Cell & Environment 37, 573–588. 10.1111/pce.12178 23941462

[CIT0049] Teh BT, Lim K, Yong CH, et al. 2017 The draft genome of tropical fruit durian (*Durio zibethinus*). Nature Genetics 49, 1633–1641. 28991254 10.1038/ng.3972

[CIT0050] Wang X, Wang H, Wang J, et al. 2011 The genome of the mesopolyploid crop species *Brassica rapa*. Nature Genetics 43, 1035–1039. 21873998 10.1038/ng.919

[CIT0051] Windsor AJ, Schranz ME, Formanová N, Gebauer-Jung S, Bishop JG, Schnabelrauch D, Kroymann J, Mitchell-Olds T 2006 Partial shotgun sequencing of the *Boechera stricta* genome reveals extensive microsynteny and promoter conservation with Arabidopsis. Plant Physiology 140, 1169–1182. 16607030 10.1104/pp.105.073981 PMC1435815

[CIT0052] Xiao H, Jiang N, Schaffner E, Stockinger EJ, van der Knaap E 2008 A retrotransposon-mediated gene duplication underlies morphological variation of tomato fruit. Science 319, 1527–1530. 18339939 10.1126/science.1153040

[CIT0053] Yang R, Jarvis DE, Chen H, et al. 2013 The reference genome of the halophytic plant *Eutrema salsugineum*. Frontiers in Plant Science 4, 46. 23518688 10.3389/fpls.2013.00046 PMC3604812

[CIT0054] Zhang Q, Chen W, Sun L, et al. 2012 The genome of *Prunus mume*. Nature Communications 3, 1318. 10.1038/ncomms2290 PMC3535359 23271652

