# Supplementary material for: Duplication and functional divergence of a calcium sensor in the Brassicaceae
Source: J Exp Bot. 2020 Jan 28;71(9):2782–95. doi: 10.1093/jxb/eraa031 (PMC7210777; doi:10.1093/jxb/eraa031)
Supplement: eraa031_suppl_Supplementary_Figures_S1-S3_and_Tables_S1-S2 [file eraa031_suppl_supplementary_figures_s1-s3_and_tables_s1-s2.pdf]

Supplementary Data

Duplication and functional divergence of a calcium sensor in the Brassicaceae

Monihan SM, Magness CA, Ryu C-H, McMahon MM, Beilstein MA, Schumaker KS

The following Supporting Information is available for this article:

Supplementary Fig. S1 Specificity of *CBL10* gene expression in transgenic Arabidopsis

Supplementary Fig. S2 Transposable elements may have mediated the *CBL10* duplication

Supplementary Fig. S3 Differences in CBL10 function reside in the amino-terminus

Supplementary Table S1 Ratio of species growth in the absence and presence of salt

Supplementary Table S2 Primers

Supplementary Fig. S1

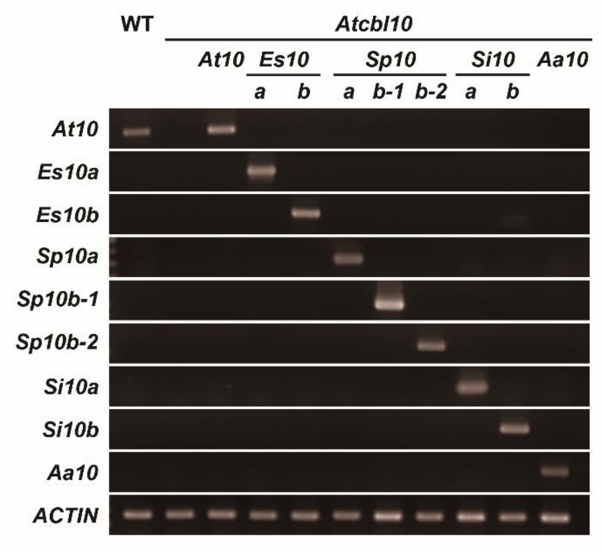

Specificity of *CBL10* gene expression in transgenic Arabidopsis. Each *CBL10* gene was expressed in the *Atcbl10* mutant under the control of the Cauliflower Mosaic Virus 35S promoter. RNA was isolated from two-week-old seedlings. Gene/species-specific primers amplify a single *CBL10* gene from each species. One representative line for each *CBL10* gene is shown. *AtCBL10* (*At10*), *EsCBL10a* (*Es10a*), *EsCBL10b* (*Es10b*), *SpCBL10a* (*Sp10a*), *SpCBL10b-1* (*Sp10b-1*), *SpCBL10b-2* (*Sp10b-2*), *SiCBL10a* (*Si10a*), *SiCBL10b* (*Si10b*), and *AaCBL10* (*Aa10*). *ACTIN*, loading control.

Supplementary Fig. S2

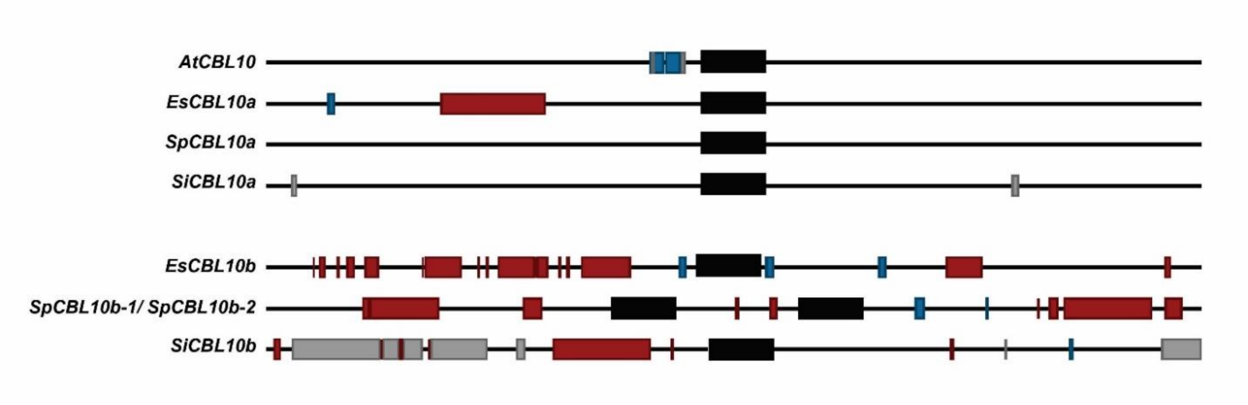

Transposable elements may have mediated the *CBL10* duplication. Black boxes, *CBL10* genes; blue boxes, DNA transposons; red boxes, retroelements; gray boxes, unknown sequence.

Supplementary Fig. S3

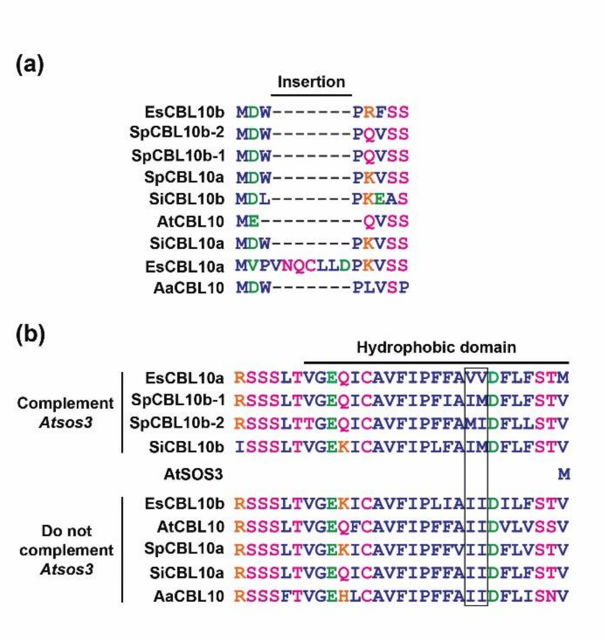

Differences in CBL10 function reside in the amino-terminus. Amino acids from CBL10 and AtSOS3 proteins were aligned and color coded based on side chain properties. Blue, non-polar side chains; magenta, polar side chains; green, negatively charged side chains; orange, positively charged side chains. Insertion, insertion of seven amino acids in the amino-terminus of EsCBL10a; hydrophobic domain, hydrophobic residues. Chimeric EsCBL10a and EsCBL10b proteins were generated and the region of the protein important for activation of the SOS pathway (a) and complementation of *Atsos3* (b) was identified (Monihan et al., 2019). (a) Brassicaceae CBL10 proteins are organized based on ability to activate the SOS pathway in yeast (Fig. 6). (b) Brassicaceae CBL10 and AtSOS3 proteins are organized based on ability to complement the *Atsos3* salt-sensitive phenotype (Fig. 7). Black box, amino acids that might underlie complementation.

Supplementary Table S1

Primers		
**Transcript**	**Forward primer**	**Reverse primer**
**Analysis of endogenous transcript levels in Brassicaceae**		
AtCBL10	GCGCCATGGAACAAGTTTCCTCTAGAT	GGCGGTACCGTCTTCAACCTCAGTGTTGAAT
EsCBL10a	GCGCTCGAGATGGTCCCGGTTAATCAATG	GGCGGATCCCGGTCTTCAACCTCTGTGTTGA
EsCBL10b	GCGCTCGAGATGGACTGGCCCAGATTTTCC	GGCGGATCCCGGTCTTCAACCTCAGTATTGA
SpCBL10a	CGTCAAGGTCTTCTAAGAGC	AGTAGCCAAGCTGGTCAAAT
SpCBL10b-1	GAAGATAAAGGGATTGCCTTTG	CTTACAATTAATACGTATACCTTGGA
SpCBL10b-2	CAAAGTGACTTACTCCGTTTGT	GTGAAAAGATTACCGGATGTTC
SiCBL10a	CACCAATCTCTGAGGACAACGAAG	GAGAGATAACCGGTGCTGT
SicBL10b	CGTTGTGAACTGATTGATACAG	CAGAAACGGTTGTTCCCAG
AaCBL10	GCGAACGCAAACGCCTAAAAC	TTTGTCCTCAATTGGTGCAGAAG
ACT2 (all species)	GAACCACCGATCCAGACACT	GGAATCCACGAGACGACCTA
**Enriching for specific *SpCBL10* transcript (UTR and CDS)**		
SpCBL10a	CGTCAAGGTCTTCTAAGAGC	AGTAGCCAAGCTGGTCAAAT
SpCBL10b-1	GAAGATAAAGGGATTGCCTTTG	CTTACAATTAATACGTATACCTTGGA
SpCBL10b-2	CAAAGTGACTTACTCCGTTTGT	GTGAAAAGATTACCGGATGTTC
**Cloning for expression in *Atcbl10* and *Atsos3***		
SpCBL10a	GCGCTCGAGATGGACTGGCCGAAAGTTTCC	GGCGGATCCCGGTCTTCAACCTCAGTGTTG
SpCBL10b-1	GCGCTCGAGATGGACTGGCCTCAAGTTTCC	GGCGGATCCCGGTCTTCCACCCCAGTGTTG
SpCBL10b-2	GCGCTCGAGATGGACTGGCCCCAAGTTTCC	GGCGGATCCCGGTCTTCAACCCCAGTGTTG
SiCBL10a	GCGCTCGAGATGGACTGGCCGAAAGTTTCC	GGCGGATCCCGGTCTTCAACCTCAGTGTTG
SiCBL10b	GCGCTCGAGATGGACTTGCCGAAAGAAGCC	GGCGGATCCCGGTCTTCAACCCCAGTGTTG
AaCBL10	GCGCTCGAGATGGATTGGCCACTAGTTTCC	GGCGGATCCCGATCTTCAACTTCAGTATTGA
**Analysis of transcript levels in *Atcbl10*, *Atsos3*, and Yeast**		
AtCBL10	TGCCGCAGCACATCGCCTCGG	AATCTGCATCAGCAAATGTTTTATC
EsCBL10a	GGTTAATCAATGTCTCCTGGAC	TTCTTCAATAAGGCGGGATG
EsCBL10b	CCTCTAGATCCAGTTCTTTG	TGGGAATGCTGTTGTCACATCC
SpCBL10a	ATATGTCAGAAAGTGGATCTGT	TCATAGAGCCTAAATGCAAA
SpCBL10b-1	CGAATCTGGCTCGTCTTGCCCTTG	CTAGGATGTTTATGCACATACG
SpCBL10b-2	CCGACATGCCGACACACGGAC	CAATAAGCCGGGATGCTTAAG
SiCBL10a	TTGATTCACAAGGAAGAGCTT	CGATCTTCTCCTCGATAGGC
SiCBL10b	CTTGCCGAAAGAAGCCTCTAT	AATCTGCATCAGCAAATGTTTTATC
AaCBL10	GCGAACGCAAACGCCTAAAAC	TTTGTCCTCAATTGGTGCAGAAG
AtCBL10	GGCTTGATTCACAAGGAAGA	TCATAGAGCCTAAATGCAAA
EsCBL10a		
EsCBL10b		
AtCBL10	TTTGTTCGATGAAAAGAAGA	TCATAGAGCCTAAATGCAAA
SpCBL10a		
SpCBL10b-1		
SpCBL10b-2		
AtCBL10	TTGATTCACAAGGAAGAGCTT	AATCTGCATCAGCAAATGTTTTATC
SiCBL10a		
SiCBL10b		
ACTIN2	GTCGTACAACCGGTATTGTG	GAGCTGGTCTTTGAGGTTTC
18SrRNA	AAACGGCTACCACATCCAAG	CCTCCAATTGTTCCTCGTTA
**Cloning for expression in yeast**		
SpCBL10	GCGCTCGAGATGGACTGGCCGAAAGTTTCC	TTTGCGGCCGCTCAGTCTTCAACCTCAGTGTTGAAG
SpCBL10b-1	GCGCTCGAGATGGACTGGCCTCAAGTTTCC	TTTGCGGCCGCTCAGTCTTCCACCCCAGTGTT
SpCBL10b-2	GCGCTCGAGATGGACTGGCCCCAAGTTTCC	TTTGCGGCCGCTCAGTCTTCAACCCCAGTGTTGAATA
SiCBL10a	GCGCTCGAGATGGACTGGCCGAAAGTTTCC	TTTGCGGCCGCTCAGTCTTCAACCTCAGTGTTG
SiCBL10b	GCGCTCGAGATGGACTTGCCGAAAGAAGCC	TTTGCGGCCGCTCAGTCTTCAACCCCAGTGTT
AaCBL10	GCGCTCGAGATGGATTGGCCACTAGTTTCC	TTTGCGGCCGCTCAATCTTCAACTTCAGTAT

Supplementary Table S2 Ratio of species growth in the absence and presence of salt

	50 mM NaCl		100 mM NaCl		150 mM NaCl		300 mM NaCl	
	**Mean**	**Group**	**Mean**	**Group**	**Mean**	**Group**	**Mean**	**Group**
**Schrenkiella**	97.591	A	60.564	A	52.363	A	25.518	A
**Sisymbrium**	106.783	A	59.753	A	35.462	A	20.673	AB
**Eutrema**	88.266	A	62.674	A	36.806	A	17.087	B
**Arabidopsis**	54.521	B	20.703	B	11.409	B	4.569	C
**Aethionema**	45.177	B	8.09	B	5.308	B	3.043	C

Results are from a Friedman's analysis (non-parametric, two-way ANOVA) of salt/control ratios for each of the salt treatments. F was significant in each and 5% Tukey mean separations are presented. Mean values with the same letter are not significantly different.
